# Prospective evaluation of GFAP point-of-care testing for rapid diagnosis of glioblastoma

**DOI:** 10.1007/s11060-026-05479-6

**Published:** 2026-02-28

**Authors:** Kristaps Blums, Love-Preet Kalra, Ntenis Nerntengian, Sabina Zylyftari, Sebastian Luger, Jens Wehinger, Deepak Bos, Stephan Barthelmes, Oliver Sakowitz, Stephan Meckel, Hansjörg Baum, Matthias Ulmer, Christian Foerch

**Affiliations:** 1 Department of Neurology, RKH Klinikum, Posilipostr. 4, 71640 Ludwigsburg, Germany; 2 Department of Neurosurgery, RKH Klinikum, Ludwigsburg, Germany; 3 Institute of Diagnostic and Interventional Neuroradiology, RKH Klinikum, Ludwigsburg, Germany; 4 Institute for Laboratory Medicine and Transfusion Medicine, RKH Regionale Kliniken Holding und Services GmbH, Ludwigsburg, Germany; 5 Department of Internal Medicine, Cancer Center, RKH Klinikum, Ludwigsburg, Germany; 6 Department of Neurology, Goethe University Frankfurt, University Hospital, Frankfurt am Main, Germany; 7 Faculty of Medicine, Goethe University Frankfurt, Frankfurt am Main, Germany

**Keywords:** GFAP, Glial fibrillary acidic protein, Biomarker, Diagnosis, Glioblastoma, Metastasis, Brain tumor

## Abstract

**Purpose:**

Glioblastoma is the most common and the most aggressive malignant primary brain tumor. The diagnosis is made by histopathological analysis following stereotactic biopsy or tumor resection. However, rapid diagnostic clarity is often required before histology becomes available. Glial fibrillary acidic protein (GFAP) has been studied as a blood biomarker for glioblastoma using conventional immunoassays. The aim of this study was to test the diagnostic value of a GFAP point-of-care device to differentiate glioblastoma from other brain tumors.

**Methods:**

Patients admitted to our hospital with a newly diagnosed intracranial lesion suspicious for a brain tumor were enrolled prospectively. Blood samples were collected prior to diagnostic and therapeutic procedures. Plasma GFAP measurements were performed using the TBI plasma test on the i-STAT Alinity^®^ (Abbott) device. The gold standard was the final histopathological diagnosis.

**Results:**

We prospectively enrolled 101 patients (mean age 63 ± 17 years, 44.6% female). GFAP plasma levels were significantly higher in patients with glioblastoma (*n* = 37; median 610 pg/mL [interquartile range 263.5–1877.5]) compared to other tumors (*n* = 64; 81.5 pg/mL [40.3–197.3]; *p* < 0.001). A cut-off point of 191 pg/mL revealed a sensitivity of 84% and a specificity of 75% for diagnosing glioblastoma. A GFAP plasma concentration of > 1000 pg/ml was indicative of glioblastoma with a specificity of 95% and a positive predictive value (PPV) of 82%. Excluding lymphomas further increased the specificity to 96% and PPV to 93%.

**Conclusion:**

GFAP measurements using a point-of-care device indicate glioblastoma with high diagnostic accuracy. GFAP testing has the potential to streamline the work-up of brain tumors.

## Introduction

Glioblastoma is an aggressive primary central nervous tumor and is the most common primary malignant brain tumor in adults [1]. It has a poor prognosis [1] and a speedy work-up is critical to provide appropriate therapy. While imaging techniques such as cranial CT and MRI are essential in establishing a suspected diagnosis of a cerebral malignancy, etiologic classification is not possible based on imaging alone [2]. A definitive diagnosis (“integrated diagnosis”) relies on extensive histomolecular evaluation of tumor tissue derived from invasive biopsy or surgery [3]. This process can take up to several weeks and often includes transports to a specialized laboratory. Furthermore, in cases of ineligibility for surgery (e.g. in elderly or multimorbid patients) a definite diagnosis cannot be established.

Glial fibrillary acidic protein (GFAP) is specific to cerebral tissue and is found in high amounts in astrocytes and ependymal cells [4]. It has a role in maintaining the cell’s cytoskeleton [5] and has shown promise as a biomarker of cerebral hemorrhage, traumatic brain injury and other diseases [6]. Glioblastomas show strong overexpression of GFAP [4]. Thus, it has also been investigated as a potential blood biomarker for glioblastoma. Several studies described increased serum GFAP concentrations in glioblastoma patients in comparison to patients with other cerebral malignancies [7–14]. In 2022 a meta-analysis of 10 studies investigating GFAP as a biomarker for glioblastoma was published by van Asperen et al. [15]. It confirmed a significant difference in GFAP serum concentration between glioblastoma patients and patients with other types of cerebral malignancies.

Whereas previous studies have investigated GFAP blood levels using sandwich ELISA immunoassays in specialized off-site laboratories, a point-of-care test for determining plasma GFAP concentrations within 15 min has been introduced in 2022 (i-STAT Alinity^®^) [16]. This point-of-care device is approved for the diagnosis of traumatic brain injury (in combination with UCH-L1, a marker for neuronal injury) [16]. In patients with a suspected brain tumor, this test could improve and streamline the standard of care pending a final histopathological diagnosis.

To date, no study has been published dedicated to the evaluation of GFAP as a biomarker for glioblastoma using a point-of-care testing device. The goal of this study was to prospectively evaluate the diagnostic accuracy of GFAP plasma levels for distinguishing glioblastoma from other brain tumors.

## Methods

### Patients

**Fig. 1:**
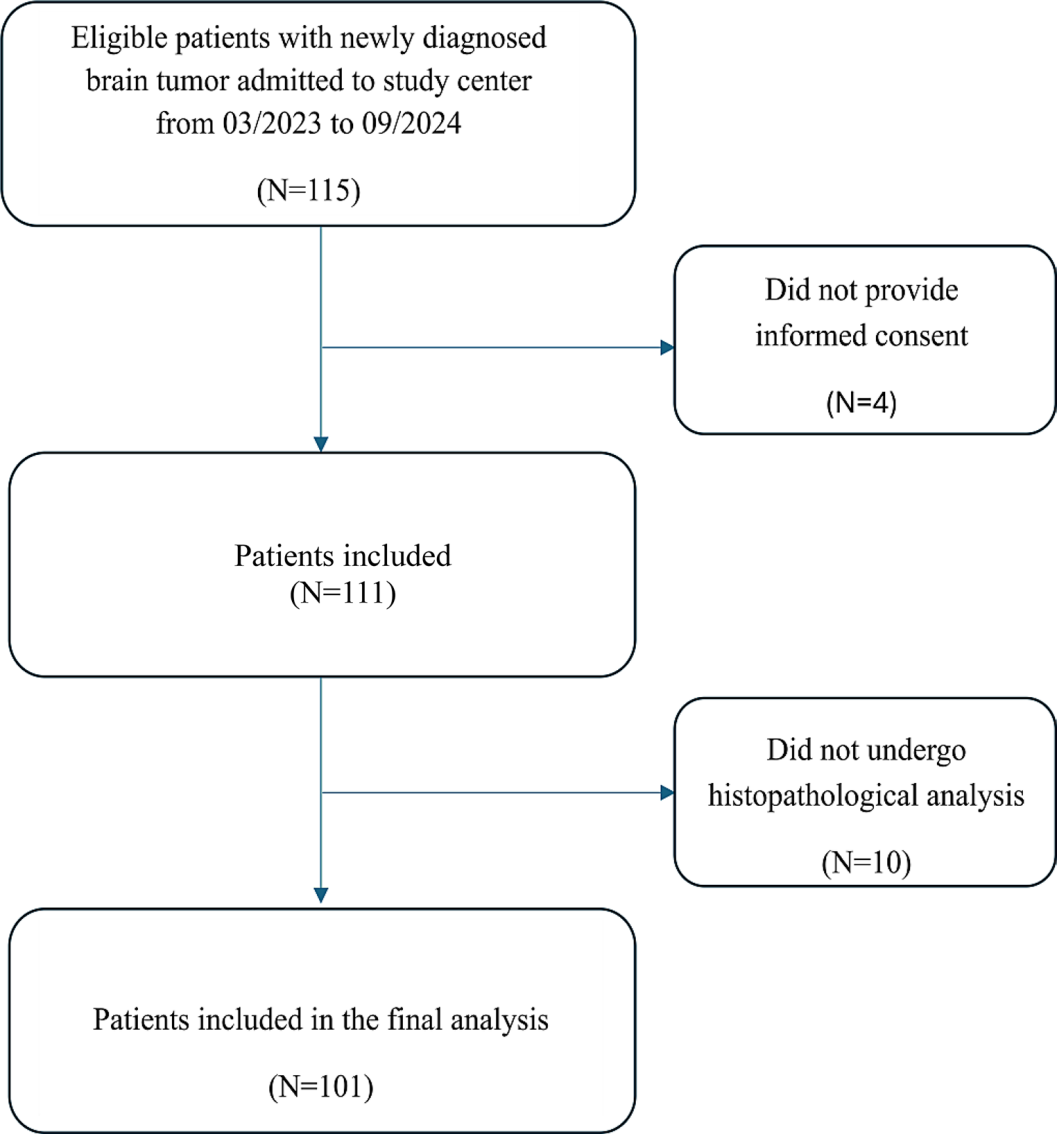
Flow diagram of the study

We prospectively recruited patients with suspected brain tumors admitted to the Department of Neurology or Neurosurgery at our tertiary care center between March 2023 and September 2024. The hospital functions as a cancer center supplying > 1 million inhabitants in the county district northern Baden-Wuerttemberg. The inclusion criteria were as follows: (1) a newly diagnosed single or multifocal space-occupying lesion on MR-imaging suspicious for a brain tumor, and (2) age of 18 years or older. The exclusion criteria encompassed the occurrence of a stroke, a traumatic brain injury, or a surgical intervention involving the central nervous system within a 3-month timeframe preceding the testing. Because the study team was not continuously present in the hospital during the recruitment period (e.g. weekends, public holidays) not every potentially eligible patient underwent screening for study inclusion. Patients who failed to consent to participation and patients who did not undergo a diagnostic biopsy or resection (i.e., lacking a final integrated diagnosis) were excluded (Fig. 1). The ethics committee of the medical association of Baden-Württemberg approved the study protocol (03/2023, F-2022-138).

The following clinical baseline variables were recorded: age, sex, Karnofsky performance status scale (KPS) (0 to 100) [10], date and type of surgical procedure performed (biopsy or resection), final histopathological diagnosis (including date of availability of results), and corticosteroid dosage (if applied). Additionally, we documented the initial MRI characteristics, including tumor localization, presence of contrast enhancement (present or not present), level of necrotic area as the fraction of the space occupying lesion (approximately 1/3, 2/3 or 3/3), presence of edema in MRI (minimal edema: +, moderate edema: ++, severe edema: +++, no edema present) [10].

### Sample collection and GFAP measurements

A single EDTA sample was collected by venous puncture prior to any invasive procedure or treatment (apart from corticosteroids) of the brain tumor. When available, the EDTA sample from routine laboratory testing at admission was used, otherwise an additional EDTA sample was drawn for study purposes. GFAP concentrations were determined immediately afterwards using the i-STAT TBI ^®^ plasma test cartridge on the Abbott i-STAT Alinity ^®^ point-of-care device. For doing so, the sample was centrifuged for 10 min at 2000 rpm. Approximately 20 µl of the resulting plasma were added to the test cartridge, which was then inserted into the i-STAT Alinity ^®^ device (measurement time: 15 min). The lowest concentration of GFAP measurable with this test is 30 pg/mL and the maximum concentration is 10,000 pg/mL. We substituted all values displaying below the lower limit of quantification on the device (i.e., < 30 pg/mL) by the value of 15 pg/mL for subsequent analysis. The histopathological results were not known at the time of testing in all cases.

Since GFAP is a cytoskeletal protein, that is not actively secreted, its plasma concentration in healthy individuals is known to be very low. In the regulatory approval study for the i-STAT Alinity^®^ (Abbott) 255 healthy subjects showed a mean plasma GFAP concentration of 19,0 pg/ml [95% confidence interval (CI) 2–51 pg/ml] [16]. Based on this information we compared the GFAP levels of GBM patients with non-GBM patients as opposed to a separate group of healthy controls.

### MR imaging

A volumetric analysis was performed by manually segmenting the tumor on thin-slice (1 mm) gadolinium-enhanced T1-weighted MRI images using the SmartBrush tool in the Brainlab Elements software (Brainlab AG, Munich, Germany) [17, 18]. For tumors without gadolinium enhancement, segmentation was based on native thin-slice MRI sequences (1 mm thick FLAIR and T2-weighted images), in which the tumor margins could be adequately identified [17]. In patients with multiple lesions, the total tumor volume was calculated as the sum of all individual tumor volumes.

### Statistical analysis

Statistical analysis was performed using IBM SPSS Statistics for Mac and Windows, version 30.0 and 31.0 (IBM Corp., Armonk, N.Y., USA). Since GFAP plasma concentrations were not normally distributed we performed statistical comparisons using the nonparametric Mann-Whitney U test. The correlation analysis was performed using Spearman’s Rho. The optimal cut-off point for differentiating between glioblastoma and non-glioblastoma patients was determined using receiver operating characteristics (ROC) curve analysis, from which the area under the curve (AUC) was determined. Sensitivity, specificity, positive predictive value (PPV) and negative predictive value (NPV) was calculated using cross tabulations. The significance level for the tests was set to 0.05.

**Table 1:** Baseline clinical characteristics of the study population, GFAP plasma levels, and type of procedure used to establish the diagnosis (B biopsy, R resection) stratified according to the histopathological diagnosis. MS=multiple sclerosis

Histology	*N*	Females (%)	Age (± SD)	Procedure (B/*R*)	GFAP pg/mL (median [IQR])
All Patients	101	44.6	63.0 ± 16.9	B:24 / R: 77	176.0 [50.0-534.5]
Glioblastoma	37	45.9	70.2 ± 11.1	B:12 / R: 25	610.0 [263.5-1877.5]
Astrocytoma					
Pilocytic astrocytoma Grade 1	4	75.0	26.0 ± 9.5	B:0 / R:4	70 [45.5-199.5]
Grade 2	5	20.0	43.0 ± 15.3	B:1 / R:4	15.0 [15.0-41.5]
Grade 3	6	16.7	40 ± 14.0	B:1 / R:5	54.0 [15.0-582.5]
Grade 4	1	0	33	B:0 / R:1	438.0
Oligodendroglioma					
Grade 2	2	100	50 ± 9.9	B:0 / R:2	39.0
Grade 3	1	100	52.0	B:0 / R:1	475.0
Metastasis	23	52.5	66.4 ± 11.6	B:0 / R: 23	98.0 [59.0-201.0]
Meningioma	5	80.0	66.4 ± 19.8	B:0 / R: 5	49.0 [15.0-154.5]
Lymphoma	10	30.0	73.8 ± 7.8	B:7 / R: 3	150.5 [104.8-515.5]
Sarcoma	2	50	73.0 ± 18.4	B:0 / R:2	158.0
Pineocytoma	1	100	57.0	B:0 / R:1	15.0
Inflammatory MS lesion	1	0	53.0	B:1 / R:0	15.0
Abscess	1	0	69.0	B:1 / R:0	72.0
Diffuse midline glioma	1	0	46.0	B:1 / R:0	50.0

## Results

We prospectively screened 115 patients presenting with newly diagnosed intracranial lesions suspicious for brain tumor (Fig. 1). Four patients refused to sign informed consent form, leaving 111 patients for study inclusion. 10 patients did not undergo histological evaluation of the intracranial lesion during the diagnostic work-up. We excluded these cases from the final analysis (Fig. 1). Subsequently, 24 patients underwent a biopsy and 77 patients underwent surgery. Mean age of the remaining 101 patients included in the final analysis was 63.0 ± 16.9 years and 44.6% were females. The baseline characteristics of the study population are shown in Table 1.

Regarding the final diagnosis after histopathological evaluation, 37 patients had glioblastoma (age 70.2 ± 11.1 years), 18 patients had low grade glioma (including astrocytoma CNS [central nervous system] WHO [world health organization] grade 1–3 and oligodendroglioma CNS WHO grade 2–3) (age 39.4 ± 14.3 years), 10 patients had lymphoma (age 73.8 ± 7.8 years), 23 patients had metastasis (age 66.4 ± 11.6 years), 5 patients had meningioma (age 66.4 ± 19.8 years), and 2 patients had sarcoma (age 73.0 ± 18.4 years). One patient had an IDH-mutated astrocytoma CNS WHO Grade 4 (age 33 years). Single cases included a pineocytoma, a diffuse midline glioma, a neurinoma, an abscess and an inflammatory lesion caused by multiple sclerosis (MS) (Table 1). The median time elapsed between determination of the GFAP test results and the availability of the final histomolecular results were 11 days (interquartile range [IQR] 8–20).

**Fig. 2:**
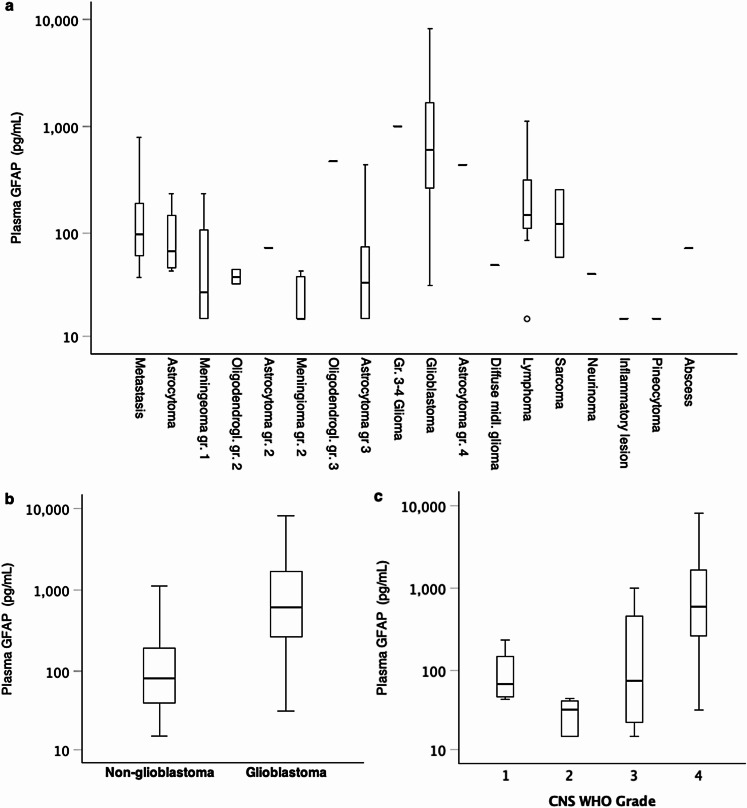
Box plots showing the distribution of GFAP plasma levels (**a**) Across different diagnoses. (**b**) In cases with and without glioblastoma and (**c**) According to the CNS WHO Grade. GFAP=glial fibrillary acidic protein, CNS = central nervous system, WHO = world health organization

Median [IQR] GFAP levels were significantly higher in patients with glioblastoma compared to patients with other tumor diagnoses (610.0 pg/mL [263.5–1877.5] vs. 81.5 pg/mL [40.3–197.3], *p* < 0.001). Median [IQR] GFAP concentrations for patients with low-grade glioma was 44 pg/mL [15.0-126.5] (Fig. 2).

**Fig. 3:**
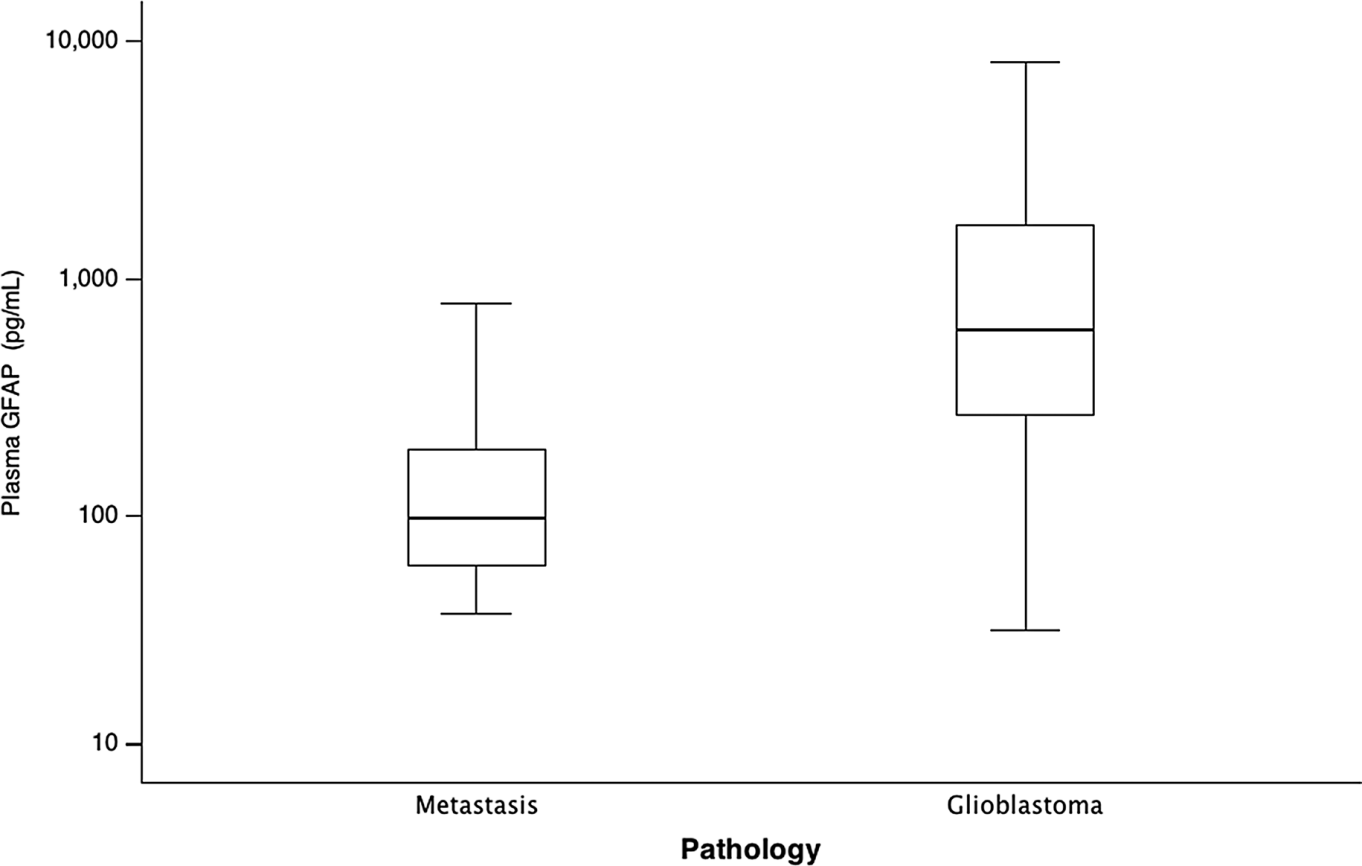
Box plots showing GFAP plasma concentrations for patients with cerebral metastases versus glioblastoma. GFAP = glial fibrillary acidic protein

Patients with lymphoma (*N* = 10) had slightly increased GFAP values (150.5 pg/mL [104.8–515.5]. Based on imaging characteristics, cerebral metastasis is a major differential diagnosis of glioblastoma. Plasma GFAP levels in glioblastoma patients were significantly more elevated compared to patients with metastases (610 pg/mL [263.5–1877.5] vs. 98 pg/mL [59.0–201.0], *p* < 0.001) (Fig. 3).

**Fig. 4:**
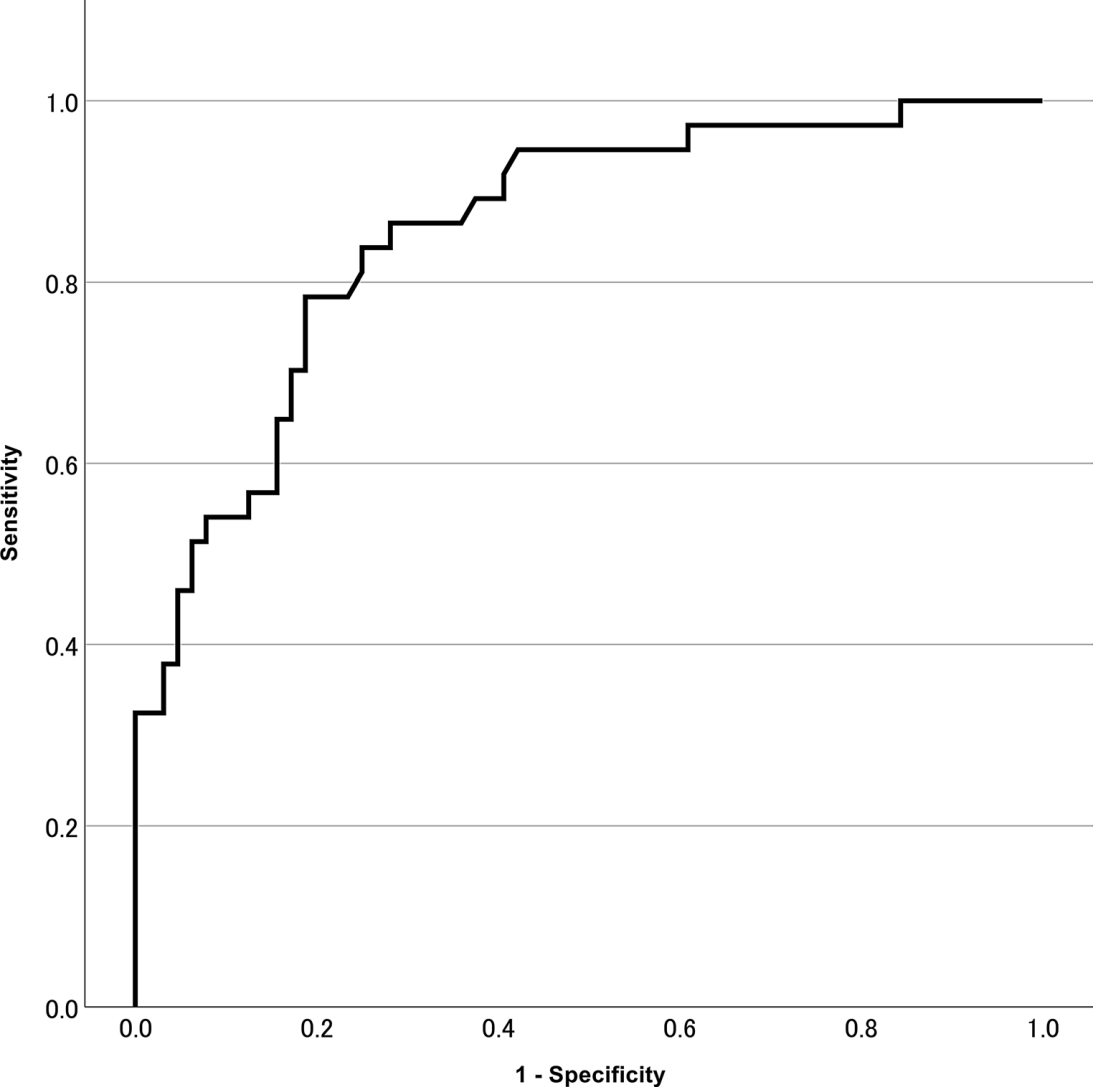
ROC analysis for differentiating glioblastoma from non-glioblastoma brain tumors. ROC = Receiver operating characteristics

ROC-Analysis revealed an optimal cut-off of ≥ 191 pg/mL for the differentiation between glioblastoma and non-glioblastoma patients [AUC 0.856, *p* < 0.001, 95% CI 0.781–0.931] (Fig. 4). Cross tabulations revealed a sensitivity of 84% and a specificity of 75% (PPV of 66%, NPV of 89%). Since CNS lymphoma may be identified and distinguished from glioblastoma by experienced radiologists using MR imaging [19], we reperformed the statistical analysis after excluding lymphoma cases (*n* = 10). In all but one case the radiology report described a suspected lymphoma (1 out of 10). By excluding the lymphoma cases PPV improved to 70%, while sensitivity, specificity and NPV did not change significantly (84%, 76% and 87%, respectively).

Since no metastasis had a GFAP level exceeding this value, we assessed an alternative cut-off point of 1000 pg/mL. With GFAP levels exceeding 1000 pg/mL the specificity was found to be 96%, while the sensitivity was reduced to 38% (PPV 82%, NPV 73%). Exclusion of the lymphoma cases led to a further increase of specificity to 98% and of the PPV to 93%, while sensitivity remained at 38% with an NPV of 69%. A lower cut-off of GFAP level of 100 pg/mL revealed a sensitivity of 92% with a specificity of 59% (PPV 57%, NPV 93%).

**Fig. 5:**
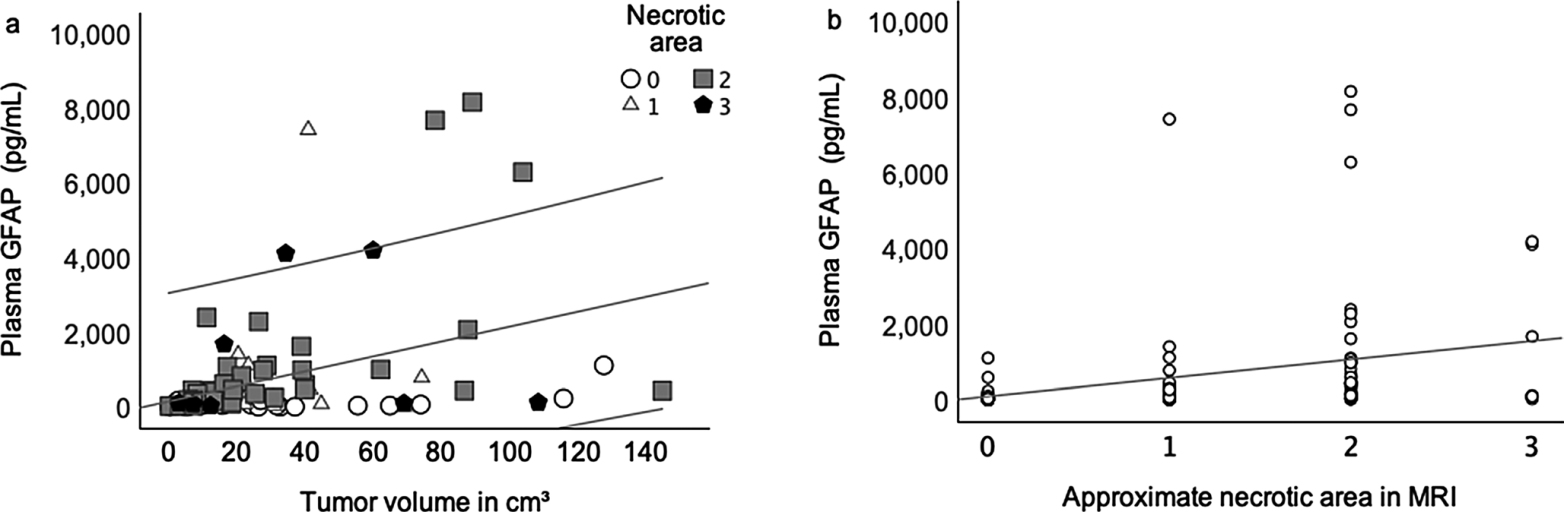
Scatter plots depicting the correlation between GFAP plasma level (**a**) and tumor volume (Spearman’s Rho, *r* = 0.44, *p* < 0.001) and (**b**) approximate necrotic area in MRI (Spearman’s Rho, *r* = 0.49, *p* < 0.001)

There was significant correlation between tumor volume and plasma GFAP level (Spearman’s Rho, *r* = 0.44; *p* < 0.001). Furthermore, the approximate tumor necrosis area correlated significantly with GFAP (Spearman’s Rho, *r* = 0.49; *p* < 0.001) as seen in Fig. 5. Out of the 4 glioblastomas that showed the most extensive necrosis, only one had low GFAP levels and the rest exhibited high GFAP levels. The highest GFAP levels were to be seen with moderate to high necrosis. GFAP levels correlated with age (Spearman’s Rho, *r* = 0.36; *p* < 0.001), mainly driven by the higher mean age of glioblastoma patients compared to patients with other tumor diagnoses. Within the cohort of glioblastoma patients, this correlation was no longer significant (Spearman’s Rho, *r* = 0.07, *p* = 0.672). Only two patients with glioblastoma did not have contrast enhancement on MRI imaging, in one of them the GFAP concentration was markedly increased (4120 pg/mL), the other one showed a GFAP concentration under the cut-off with 117 pg/ml.

## Discussion

Our prospective study demonstrated increased GFAP levels in glioblastoma as compared to other intracranial space-occupying lesions when measured with a point-of-care device. GFAP is expressed in astrocytes and – in particularly high quantities – in malignant gliomas [12, 20–22]. Its release in the bloodstream is facilitated by tumor cell necrosis and an impairment of blood-brain-barrier, both common in glioblastoma. Previous studies exploring GFAP in glioblastoma patients were obtained through ELISA testing after continuous sampling and long-haul storage procedures [6–15]. The immediate on-site availability and ease of implementation increases the feasibility of GFAP testing significantly, since the point-of-care testing can be done with minimal training and the results are available in 15 min, without relying on specialized laboratories and avoiding delays caused by transportation and time-consuming laboratory testing.

The optimal sensitivity and specificity as revealed by ROC-analysis was achieved with a GFAP cut-off value of 191 pg/mL. Only three of the 37 glioblastomas in our study showed GFAP levels below 100 pg/ml. One of these 3 patients had a very small tumor volume of 0.5 cm^3^. In this case the amount of astroglial necrosis is likely too small to build up a detectable plasma GFAP level. In general, we found a strong association between GFAP and glioblastoma volume as well as between GFAP and the necrotic area on MR imaging (Fig. 5), consistent with previous research [9, 12, 23]. None of the subjects with glioblastoma showed a GFAP concentration under the lower limit of detection of 30 pg/ml. Previous studies described a ‘‘sensitivity gap’’ - i.e. low GFAP blood levels in patients with glioblastoma – probably resulting from a loss of GFAP expression through dedifferentiation [12]. At the cut-off of 100 pg/ml we found the effect to be modest with a sensitivity of 92% and an NPV of 93%. Hence a GFAP concentration below 100 pg/ml is unlikely to be found in a patient with glioblastoma, if the tumor volume is considerable.

Whereas patients with metastases overall showed low GFAP values, patients with lymphoma had slightly elevated values. Increased GFAP levels in CNS lymphoma have been reported in a previous study, but only in a single case [12]. One potential explanation could be that primary CNS lymphoma exhibits reactive gliosis and astrocytosis [24]. Two out of 10 patients with a final diagnosis of CNS lymphoma exhibited GFAP levels > 1000 pg/mL in our study. The strongly elevated GFAP level in the first case was most likely caused by secondary intracerebral bleeding. In the other case (located in the frontal lobe), tumor volume was as large as 127.0 cm^3^. We speculate that the excessive mass-effect in the anterior fossa was the cause of localized damage of glial cells resulting in the release of GFAP. No other space occupying lesions apart from glioblastoma and one high grade glioma (CNS WHO grade of at least 3, but not definitively graded) showed GFAP levels above 1000 pg/mL. Thus, a GFAP concentration above the cut-off value of 1000 pg/mL together with information from MR imaging (if there is no suspicion for a large CNS lymphoma) is highly suggestive of the presence of glioblastoma. Metastases on the other hand would be highly unlikely. Vice versa, a GFAP concentration of < 100 pg/mL in patients with an intracerebral space occupying lesion is indicative for the absence of glioblastoma.

GFAP point-of-care testing in patients suspicious of having a malignant brain tumor could influence clinical decision making. In one case - which was not included in the final data analysis due to lack of histopathological data - the GFAP level was above the highest measurable level of 10,000 pg/mL. This patient was 90 years old and denied any surgical intervention, forgoing standard diagnostic procedures. Considering the imaging findings and the clinical progression, alongside the highly elevated GFAP values, the most likely diagnosis of glioblastoma was made without invasive procedures and palliative care initiated. This was an exceptional case, as the histopathological evaluation remains the gold standard for diagnosis and cannot yet be replaced by GFAP testing. Nevertheless, when used together with other clinical information GFAP testing in patients with a space-occupying intracranial lesion has the potential to prevent delays in initiation of treatment. A newly diagnosed solitary cerebral space occupying lesion in a patient with an existing cancer diagnosis would be prone to be taken for a metastasis. There have been cases [25], where a glioblastoma has been assumed to be a melanoma metastasis and treatment has been initiated without a (repeat) biopsy, delaying standard glioblastoma treatment [25]. Such cases are not isolated, as the authors of this paper have encountered similar misdiagnoses. We assume that in the case of a markedly elevated plasma GFAP a decision would be made to perform a biopsy without delay as an affirming as opposed to a ruling-out measure.

Conversely, in younger patients with no history of malignancy and a newly diagnosed strongly contrast-enhancing (and thus less likely to be a low grade glioma) cerebral lesion, a very low plasma GFAP level would be suggestive for the presence of a metastasis. In such cases it would be reasonable to initiate staging CT-scans to look for a primary tumor.

The presence of a concurrent intracerebral hemorrhage (ICH) must be considered, since it has been demonstrated to increase GFAP levels as well [26]. A study on prehospital GFAP levels in acute stroke showed the median plasma GFAP concentration to be 208 pg/ml [IQR 60-5907] [26]. If a brain tumor shows secondary bleeding the GFAP level could be elevated even in the absence of a glioblastoma.

The limitations of this study include a relatively small sample size of low- and high-grade gliomas compared to glioblastomas, metastases and other diagnoses. The question, if GFAP testing can help in the diagnosis of non-contrast-enhancing glioblastoma cases, has been raised before, as underestimation of tumor grade can lead to delays in diagnosis [15]. This question cannot be answered by our study as only two glioblastoma patients lacked contrast enhancement in MRI. In one case the GFAP value was markedly increased and in the other only marginally increased (under the cut-off of 191 pg/ml). Further large prospective studies are needed to fully investigate the sensitivity and specificity of the GFAP point-of-care testing in glioblastoma patients and determine whether serum GFAP levels are increased in high-grade gliomas without contrast enhancement, as well as to evaluate its role in monitoring disease progression. The interpretation of the GFAP levels must consider imaging results as lymphomas and ICH can also have elevated GFAP levels.

## Conclusion

The findings of this prospective study indicate that GFAP levels, as measured by a point-of-care device are indicative of glioblastoma in patients with a newly diagnosed space-occupying intracranial lesion. Warranting reconfirmation in larger cohorts, this information could be used to establish glioblastoma diagnosis in cases where histomolecular evaluation is not possible (e.g. palliative setting or low Karnofsky Performance Status). Moreover, in patients with a preexisting cancer, elevated GFAP levels could elicit a speedy biopsy and help prevent a misdiagnosis. Vice versa, a low GFAP level would encourage early staging and enable faster discovery and therapy of a possible primary tumor. As a limitation it should be noted that GFAP testing is not always conclusive. Further studies are needed to clarify the role of GFAP as a marker of tumor recurrence during the therapeutical course.

## Data Availability

The data used and analyzed in this study can be obtained from the corresponding author upon reasonable request.
